# Exploring the landscape of dismantling strategies based on the community structure of networks

**DOI:** 10.1038/s41598-023-40867-2

**Published:** 2023-09-02

**Authors:** F. Musciotto, S. Miccichè

**Affiliations:** Dipartimento di Fisica e Chimica-Emilio Segrè, Università degli Studi di Palermo, Viale delle Scienze, Ed. 18, 90128 Palermo, Italy

**Keywords:** Complex networks, Statistics

## Abstract

Network dismantling is a relevant research area in network science, gathering attention both from a theoretical and an operational point of view. Here, we propose a general framework for dismantling that prioritizes the removal of nodes that bridge together different network communities. The strategies we detect are not unique, as they depend on the specific realization of the community detection algorithm considered. However, when applying the methodology to some synthetic benchmark and real-world networks we find that the dismantling performances are strongly robust, and do not depend on the specific algorithm. Thus, the stochasticity inherently present in many community detection algorithms allows to identify several strategies that have comparable effectiveness but require the removal of distinct subsets of nodes. This feature is highly relevant in operational contexts in which the removal of nodes is costly and allows to identify the least expensive strategy that still holds high effectiveness.

## Introduction

Network dismantling has become a topic of great interest in many research and operational fields. The basics of dismantling are grounded in the seminal works of Havlin^1^ and Barabasi^2^ that were the first ones to study the resilience properties of networks. These two works have greatly elucidated the resilience mechanisms of networks under random^1^ and targeted^2^ attacks. Also, they indicated how percolation-based methodologies can be fruitfully used to investigate networks properties.

Indeed, network dismantling can be seen as the reverse of network resilience, with the *trait d’union* being the percolation phenomenon. Indeed, reversing the mechanism of percolation we observe how large connected components of a network abruptly disappear through the progressive deletion of links or nodes, ranked according to some criterion^3^. Different ranking criteria have been proposed to maximize the efficiency of network dismantling, focusing on the detection of the most influential nodes to remove^4–7^, or adopting holistic approaches that focus on the collective, emergent features of complex networks^8–11^. Due to its flexibility, network dismantling has been applied to different domains, ranging from biology^12^ to socio-technical systems^13,14^ and crime^15,16^.

In Ref.^15^ we have considered a dismantling methodology that is based on the membership of mafia affiliates to specific Mafia syndicates. Specifically, we show how prioritizing the removal of nodes that have the highest number of connections with members coming from different syndicates guarantees good dismantling performances. The aim of the present work is to generalize that approach by exploring the idea of dismantling a network with a strategy modeled on its community structure. Specifically, we prioritize the removal of nodes that have the highest number of links with nodes in other communities. Indeed, these nodes are more likely to bridge different areas of the network. We observe how the community can be given (as in our previous investigation of a Mafia network), or can be detected using one of the several methods available in literature^17^. In the latter case, there is an unavoidable degree of arbitrariness in the choice of the community detection algorithm as well as to the stochastic nature inherited in most of such algorithms.

This is an issue that has been extensively investigated in recent papers^18,19^. In Ref.^18^ the community structure is used to retrieve all nodes that bridge different communities, that are then ranked for removal according to betweenness. In Ref.^19^ it is shown that community-based network dismantling significantly outperforms existing techniques in terms of solution quality and computation time in the vast majority of the analysed real-world networks, while standard dismantling techniques mainly excel on model networks. The dismantling protocol of Ref.^19^ involves the exploitation of five community detection algorithms (STEP 1). For each of them a dismantling strategy is devised (STEP 2). In fact, the final goal is to have all communities disconnected, such that no single inter-community link remains. An important concept here is the notion of a condensed community network, a representation in which each node represents a community and two communities are connected if and only if the two communities have at least one inter-community link in the original network. For the condensed community network dismantling, the devised strategy consists in attacking links. The dismantling strategy we propose is different from the one proposed in Ref.^19^. In fact, by mimicking the approach followed in Ref.^15^, we start removing nodes—rather than cutting links—based on the number of links that a node forms with other nodes outside its community. Indeed, we consider a node-percolation dismantling approach rather than a link-percolation approach. It is worth considering that our protocol assumes that the community structure and the number of links that a node forms with other nodes outside its community are extracted at each iteration, i.e. after each node removal.

We test our methodology both on synthetic networks obtained by using stochastic block models^21^ with different community structures, and on real-world networks. We compare the performance of our methodology with the following approaches:

- Interactive Degree (ID), based on the removal, at each step, of the node with the highest degree. This approach requires the calculation of degree after each removal.
- Interactive Betweenness (IB), based on the removal, at each step, of the node with the highest betweenness. This approach requires the calculation of betweenness after each removal.
- Collective Influence (CI), based on the removal, at each step, of the node with the highest collective influence. CI of node *i* is defined as $CI_{i} = (k_i-1)\sum _{j \in B_{l}^i}(k_j-1)$ where *k* is the degree and $B_l^i$ is the frontier of nodes at distance *l* from node *i*^20,23^. CI is recomputed after each removal. In what follows, we use CI with $l=2$ and $l=3$.

Our methodology outperforms methods based on ID and CI and is comparable with IB computation, especially on networks with well pronounced community structure. Conversely, our protocol is computationally much faster that protocols based on interactive betweenness computation and it is competitive with protocols based on interactive degree computation.

The community based dismantling strategy is not unique, being it dependant on the specific network partitioning considered. Rather than being a flaw, we believe that this is a strength of our methodology. In fact, since we are interested in dismantling—and not in resilience—having different dismantling paths can be an advantage in all operational situations in which a specific set of nodes to be removed might be unreachable for several reasons, mainly related to their accessibility and/or the removal costs. However, when providing different strategies, we are interested in the robustness of the dismantling efficiency—that we estimate through the established R-measure. We are therefore able to show that at least for the cases accounted for in this work, the value of R remains essentially the same on all possible strategies, notwithstanding the community detection algorithm, thus indicating that the methodology is quite robust.

Finally, we find that the performances of our strategy considerably drop when considering random networks, since our strategy becomes comparable with dismantling strategies based on interactive degree. This is again an indication of the effectiveness of our methodology, being it more performing when the community structure is genuinely significant.

The paper is structured as follows: In section “Methods” we briefly sketch the methodology. In section “Results” we apply our approach to a class of benchmarks with different fine tuned community structure and on three different sets of real world networks and in section “Discussion” we discuss the main implications.

## Methods

Resilience properties of networks have been firstly studied within the context of random attacks^1^, showing that real networks, due to their specific properties (the presence of heterogeneous degree distributions among others) are strongly robust against dismantling strategies where the order of removal is random. Conversely, it has later been shown that targeted attacks, in which nodes are removed according their centrality, are much faster in dismantling a network^2^. In both cases percolation techniques provide the theoretical background and the operative protocols for understanding how node removal affects network resilience.

Our methodology generalizes the framework of network dismantling by considering a set of removal strategies of nodes that are based on their membership in non-overlapping communities^19^. Specifically, nodes will be removed from the network starting from the nodes that have the highest number of links with nodes of other communities.

We therefore propose an iterative procedure that at each iteration involves the following four steps:

- Partition the network according to any community detection algorithm
- Select the node with the highest number of links with nodes of other communities
- Remove the selected node and its links

Starting from the original network, the iterations proceed until we are left with a network where communities can no longer be detected. This step is reached either because the network is composed of isolated nodes, or because we choose to stop when communities have sizes smaller than a certain predetermined value $S_{stop}$. Hereafter we will stop the iterations when observing communities with sizes smaller than $S_{stop}=3$.

Since at each iteration we remove a node from the existing network and thus affect the detected community structure, we reapply at each step the chosen community detection algorithm. This poses some computational constraints in terms of size of the networks as well as in terms of the community detection algorithm computational efficiency. Moreover, since community detections algorithms usually involve some level of stochasticity, we apply the whole dismantling procedure *M* times in order to assess what is the robustness of the obtained results.

In this work we will mostly consider the community detection algorithm introduced in^22^. This algorithm belong to the wide class of those algorithms that search communities by maximizing the network modularity. It provides an improvement of the Louvain algorithm^24^ because it provides (1) a better detection of communities and (2) faster computational times, which is relevant to our case given the need to rerun the community detection at each iteration of the procedure. In specific cases we will compare the results obtained using the Leiden algorithm with those obtained using the Louvain^24^ and Infomap^28^ community detection algorithms.

Our code is written in Python and we use appropriate libraries for the Leiden, the Louvain and betweenness algorithm implementations. In particular:

- we use the igraph library for computing betweenness and other network metrics^25^. The igraph implementation of betweenness is commonly recognized as one of the fastest routines.
- As for the Leiden community detection algorithm, we consider the library developed by one of the authors^26^. This is written in C++ and can be used within igraph as well.
- As for the Louvain community detection algorithm, we use the routine implemented within the igraph library.

As such, we believe that our code is not computationally biased towards one method or the other.

As a measure of the robustness to attacks we use the robustness metric *R* defined as^27^:

1 $$\begin{aligned} R={1 \over N} \, \sum _{Q=1}^N \, s(Q) \end{aligned}$$

where *N* is the number of nodes in the network and *s*(*Q*) is the fraction of nodes in the largest connected component after removing *Q* nodes. The normalization factor $N^{-1}$ ensures that the robustness of networks with different sizes can be compared. The range of possible *R* values is between $N^{-1}$ and 0.5. From the geometric point of view, *R* represents the area between the curve spanned by the fraction of nodes in the largest connected components as a function of the number of removed nodes.

As mentioned above, the only example we know of a community-based dismantling procedure is given in Ref.^19^. Of course, the two protocols are related to each other. A pedagogical example of such differences, is given in Appendix A.

## Results

### Benchmarks

**Figure 1:**
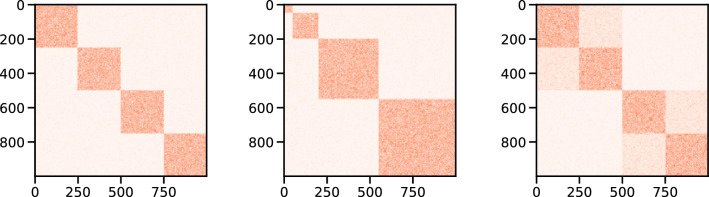
Examples of adjacency matrices of synthetic networks with 1000 nodes and (**a**) four communities of homogeneous size, 250 nodes each, (**b**) four inhomogeneous communities with [50, 150, 350, 450] nodes and (**c**) a nested community structure with 2 macro communities of 500 nodes more loosely connected and 4 smaller communities of 250 nodes more densely connected.

**Figure 2:**
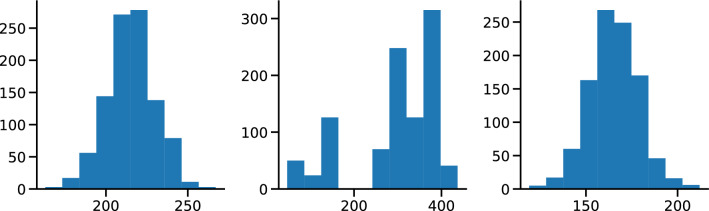
Examples of degree distributions of synthetic networks with 1000 nodes and (**a**) four communities of homogeneous size, 250 nodes each, (**b**) four inhomogeneous communities with [50, 150, 350, 450] nodes and (**c**) a nested community structure with 2 macro communities of 500 nodes more loosely connected and 4 smaller communities of 250 nodes more densely connected.

**Figure 3:**
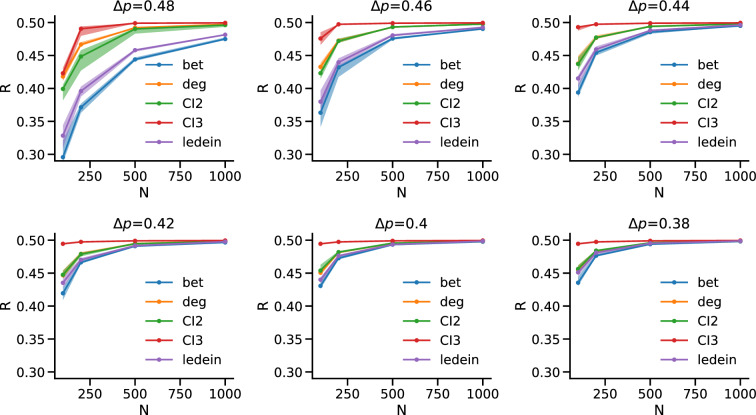
Homogenous communities. Each panel shows R as a function of *N* for ensembles of synthetic networks with homogeneous communities for a different value of $\Delta p$, which decreases from top left to bottom right from $\Delta p=0.48$ to $\Delta p=0.4$. The solid lines represent the median over 10 iterations of the methods over 100 synthetic replicas, while the shaded area is the interval between the $10{\rm th}$ and the $90{\rm th}$ percentile. We highlight that the distribution of Rs is not skewed, and median and mean almost overlap in value for all simulations.

We apply our method to a class of synthetic benchmark generated using a stochastic block modeling approach^21^ to generate well defined, statistically significant community structures. We investigate different structures, namely (1) communities of homogeneous size (Fig. 1a), (2) communities of different sizes (Fig. 1b) and (3) nested communities (Fig. 1c) to test the performance of the method in different conditions. Depending on the size and the structure of the implanted blocks, the degree distribution of the different benchmark is either poissonian or bimodal (Fig. 2).

We started from the homogeneous case and generated ensembles of networks with four communities defined by the following block probabilities, $$p_{ij} = \begin{pmatrix} 0.02+\Delta p &{} 0.02 &{} 0.02 &{} 0.02\\ 0.02 &{} 0.02+\Delta p &{} 0.02 &{} 0.02 \\ 0.02 &{} 0.02 &{} 0.02+\Delta p &{} 0.02 \\ 0.02 &{} 0.02 &{} 0.02 &{} 0.02+\Delta p \\ \end{pmatrix}$$, where *i*, *j* vary between 1 and 4 and $\Delta p$ is a parameter that quantify how much the community structure is well defined. After fixing the number of nodes *N* of the synthetic benchmark, we generated 100 networks with a given value of $\Delta p$ and for each synthetic network we applied 10 times our dismantling approach based on the communities structure detected with the Leiden algorithm^22^. We quantified the performance by means of the R-measure and took the median over all the realizations. We repeated the procedure for with *N* ranging in the interval [100, 200, 500, 1000] and $\Delta p$ in the interval [0.38, 0.48]—to study the robustness of the dismantling performance when the community structure becomes less defined—and we compared the results with ID, IB and CI based approaches (Fig. 3).

We find that, for well defined community structures (higher $\Delta p$), the dismantling approach based on interactive betweenness (IB) performs significantly better than the interactive degree (ID) one (as already confirmed in previous studies^29^), with our community based approach performing slightly worse than IB but pronouncedly better than ID. CI based approaches perform slightly better or slightly worse than ID, but always significantly worse than IB and our method. This pattern holds for different values of *N*. However, when the community structure is less defined (lower values of $\Delta p$) the difference between the three methods is less marked.

**Figure 4:**
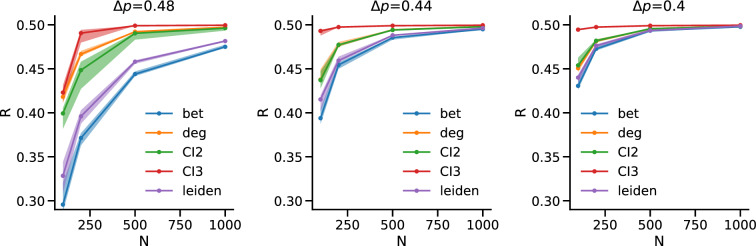
Communities of different size. Each panel shows R as a function of *N* for ensembles of synthetic networks with communities of different sizes for a different value of $\Delta p$, which decreases from left to right from $\Delta p=0.48$ to $\Delta p=0.4$. The solid lines represent the median over 10 iterations of the methods over 100 synthetic replicas, while the shaded area is the interval between the $10{\rm th}$ and the $90{\rm th}$ percentile. We highlight that the distribution of Rs is not skewed, and median and mean almost overlap in value for all simulations.

In order to check the robustness under different conditions, we repeated the analysis in the case of communities of different sizes, finding again the same pattern (Fig. 4): IB is the best dismantling method with our community based approach giving similar but slightly worse performances and ID/CI being significantly worse. The differences in performance among the three methods decrease with $\Delta p$.

We performed an additional check on ensembles of synthetic networks with a nested community structure defined by the probability matrix $$p_{ij} = \begin{pmatrix} 0.5 &{} 0.02+\Delta p_{in} &{} 0.02 &{} 0.02\\ 0.02+\Delta p_{in} &{} 0.5 &{} 0.02 &{} 0.02\\ 0.02 &{} 0.02 &{} 0.5 &{} 0.02+\Delta p_{in}\\ 0.02 &{} 0.02 &{} 0.02+\Delta p_{in} &{} 0.5 \end{pmatrix}$$, where $p_{ij}$ reproduces the adjacency matrix of Fig. 1c and $\Delta p_{in}$ tunes the nestedness of the partition: lower values create a network where the 4 micro communities are significantly more densely connected than the macro ones, while higher values dilute the nestedness in a more homogeneous structure like the one of Fig. 1a.

**Figure 5:**
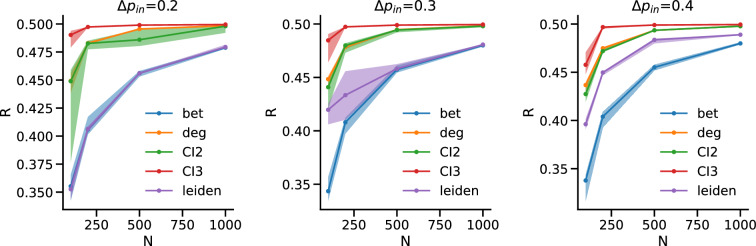
Nested communities. Each panel shows R as a function of *N* for ensembles of synthetic networks with nested communities for a different value of $\Delta p_{in}$, which increases from left to right from $\Delta p_{in}=0.2$ to $\Delta p_{in}=0.4$. The solid lines represent the median over 10 iterations of the methods over 100 synthetic replicas, while the shaded area is the interval between the $10{\rm th}$ and the $90{\rm th}$ percentile. We highlight that the distribution of Rs is not skewed, and median and mean almost overlap in value for all simulations.

We find that when $\Delta p_{in}$ is low—and thus the nestedness is more pronounced, with the macro communities being more loosely connected if compared to the micro ones—our community based method performs as well as the IB, while as expected ID performs significantly worse. When $\Delta p_{in}$ increases, the difference between IB and our method increases to the advantage of IB, but they both still perform significantly better than ID/CIs (Fig. 5).

**Figure 6:**
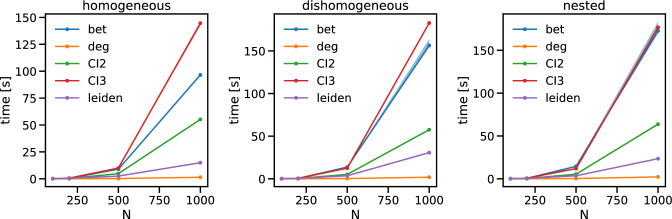
Computational complexity. Each panel shows the computational time of the three methods as a function of *N* for ensembles of synthetic networks with homogeneous communities (left panel), heterogeneous communities (center panel) and nested communities (right panel). The computation was performed on a workstation Intel$^\circledR$ Xeon$^\circledR$ Gold 5118 CPU @ 2.30GHz—2 processors— total 24 cores 512 Gb RAM. The solid lines represent the median over 10 iterations of the methods over 100 synthetic replicas, while the shaded area is the interval between the $10{\rm th}$ and the $90{\rm th}$ percentile.

As a next step, we estimated the computation complexity of the three methods for the three classes of benchmark (Fig. 6) as a function of time (we set $$\Delta p=0.48$$ for homogeneous and heterogeneous communities and $$\Delta p_{in}=0.2$$ for the nested communities). We find that IB and CI ($$l=3)$$ are the most computational demanding approaches—however, it should be noted here that we did not implement the optimized version of the CI algorithm suggested in Ref.^23^. Conversely, the ID is the least challenging method as degree is computationally cheap. We find that our methods falls in between, being approximately 5/6 times faster than IB.

### Real networks

**Figure 7:**
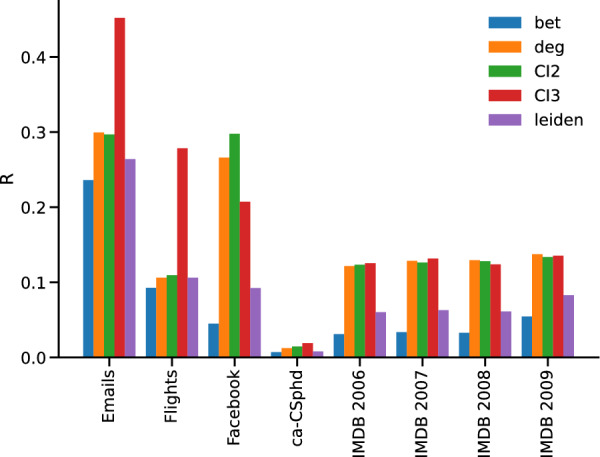
Real networks. R of different real networks.

We then applied our method to the following set of real world networks.

#### IMDB actors

We analysed the set of actors playing in movies indexed in the IMDb database (http://www.imdb.com/). IMDb is the largest web repository of world movies. We consider here the bipartite relationship between movies and actors produced in the period 1990–2009 all over the world. The set includes movies realized in 169 countries. We slice the data temporally extracting one bipartite network per year and, for each of these networks, we generate the univariate projected network of actors, by setting a link between any two pair of actors whether they played in the same movie^30^.

In what follows, we limit our analysis to male actors. We started from the networks related to the years from 2006 to 2009 and applied the IB, ID, CIs and community based dismantling approaches to all of them. We find also in this case that IB and our community based approach (implemented again using Leiden as a community detection algorithm) perform significantly better than ID/CIs, with IB achieving the best performance (right bars in Fig. 7). Again, the approaches based on CI perform similarly to ID.

In order to test how robust is our community based approach, we applied it for each of the 20 yearly networks in the interval 1990–2009 M times, with M = 30. In Table 1 we show, for each yearly network, the number of nodes (second column), the number of links (third column), the mean and standard deviations of the R measure. We observe that our methodology is quite robust, as the dispersion of the R values around their mean is below $0.5\%$ of the mean.

**Table 1:** Summary statistics of the IMDB networks of actors year by year.

Year	Nodes	Links	*R*	Year	Nodes	Links	*R*
2009	34784	431074	0.0828 ± 0.0002	1999	19915	296424	0.0601 ± 0.0003
2008	35287	463305	0.0611 ± 0.0003	1998	17848	258265	0.0672 ± 0.0005
2007	32948	455193	0.0628 ± 0.0002	1997	17256	260975	0.0739 ± 0.0004
2006	33930	472873	0.0603 ± 0.0002	1996	16451	259303	0.0658 ± 0.0005
2005	30936	443281	0.0583 ± 0.0003	1995	14918	219909	0.0652 ± 0.0004
2004	27532	362056	0.0495 ± 0.0004	1994	14373	225495	0.0673 ± 0.0004
2003	23874	352889	0.0492 ± 0.0003	1993	13855	209918	0.0644 ± 0.0003
2002	21190	309913	0.0630 ± 0.0003	1992	13907	218349	0.0581 ± 0.0002
2001	21708	313105	0.0623 ± 0.0003	1991	13626	203889	0.0601 ± 0.0004
2000	19894	292269	0.0607 ± 0.0002	1990	13402	191827	0.0646 ± 0.0004

The columns report (1) the release year of the movies on which the network has been constructed, (2) the number of nodes, (3) the number of links, (4) the R measure of the community based strategy.

**Figure 8:**
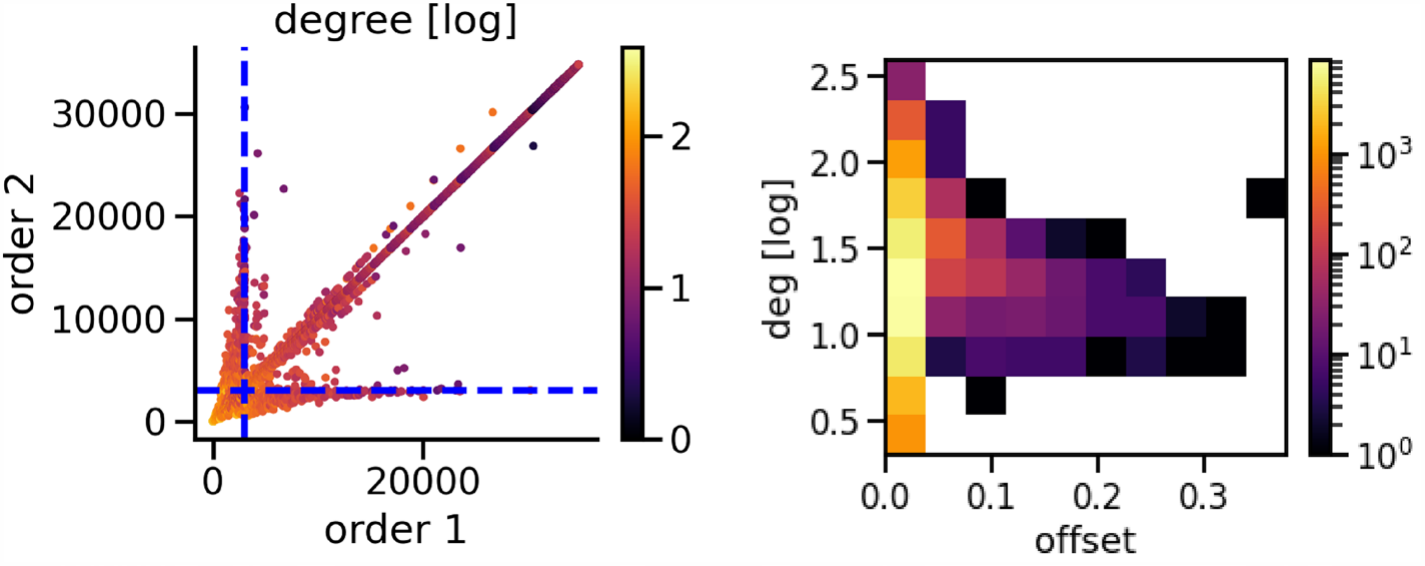
Left panel: scatter plots of the rankings of nodes in two different realizations of the dismantling procedure based on the community structure of the 2009 IMDB network. The color of nodes reflect their degree (logarithmic scale) while the two dashed blue lines represent the number of nodes that correspond to critical threshold $q_c^{(comm)}$ of network dismantling. Right panel: 2D histogram of the average offset between rankings across all couples of the *M* realizations of the dismantling procedure (x-axis) versus logarithm of the degree (y-axis). Colors represent the number of counts in each bin.

In spite of the robustness of R across several realizations, we highlight that each realization gives different sequences of node removals. Thus, our approach is able to identify several dismantling strategies that have the same effectiveness but involve different nodes. In order to highlight the difference among strategies, in the left panel of Fig. 8 we show a scatterplot reporting the node ranks obtained in two different realizations of our methodology to the 2009 network. Each node is coloured according to its log degree in the original network—the color code is shown in the palette at the right of the panel. In the figure, we highlight with blue dotted lines the two regions—the one at the left for strategy 1 and the one at the bottom for strategy 2—that contain the nodes that need to be removed to maximize the size of the second largest connected components. In fact, the number of such nodes is a possible measure of the efficiency of a dismantling approach^2,15^. If we focus on these two regions, we see that the first few nodes with high (original) degree are removed always in (almost) the same order, and indeed they all fall on the diagonal and are located in the small bottom left box. Off-diagonal dots, that represents nodes that are removed in significantly different order in the two realizations of the dismantling procedure, have usually darker colors, thus indicating that they have low degree. All the points that are in the two rectangles up and right the bottom left square represent nodes that are removed in one of the strategies but not in the other one, thus giving rise to different dismantling strategies. The left panel of Fig. 8 illustrates the comparison between only two of the $M=30$ realizations of the dismantling procedure. In order to obtain a more comprehensive view we consider, for each node in the network, the absolute value of the relative difference between the ranking positions in all couples of the *M* different applications of our procedure, which we call offset. Specifically, given two dismantling rankings $r^1$ and $r^2$, the offset *o* for a generic node *i* is defined as $o_i=\frac{\left| r^1_i-r^2_i \right| }{N}$, where *N* is the number of nodes in the network. In the right panel we report a 2D histogram with on the horizontal axis such offsets and on the vertical axis the log degree. The counts of each 2D bin of the histogram are shows through the color code reported in the palette at the right of the panel. One can easily see that larger offsets occur less frequently (darker colors) and for nodes with degree of a few units, i.e. between $e^{0.8} \approx 2$ and $e^{1.8} \approx 6$.

The above results show that the intrinsic stochasticity associated to most community detection algorithms clearly affects the sequence of the nodes that have to be removed in order to dismantle the considered network. However, the procedure remains robust with respect to its efficiency, measured in terms of R. This would suggest that indeed R refers to a peculiar property of the network rather than of the way in which we partition it. Indeed, when considering the Louvain community detection methodology^24^ we get a value of $0.0849 \pm 0.0004$ for the 2009 actors network, which is very close the one obtained with the Leiden clustering. Using Infomap^28^, we get $R_{infomap}=0.1014 \pm 0.0001$.

#### The airport network

In order to test this last hypothesis we consider in this section a smaller dataset that will be partitioned with different community detection algorithms all based on the maximization of modularity.

The network has $N=1390$ nodes and $L=9758$ symmetric links distributed along 14 connected components whole largest size is 1363. Nodes are airports of the ECAC area and links between airports are established whenever there is a flight that connects them^31–34^. Our dataset comprises all the flights that, even partly, cross the ECAC airspace for the entire 2017 year. Data were obtained by EUROCONTROL (http://www.eurocontrol.int), the European public institution that coordinates and plans air traffic control for all of Europe. Specifically, we obtained access to the Demand Data Repository (DDR)^35^ from which one can obtain all flights followed by any aircraft in the ECAC airspace. Data about flights contain several types of information. In the present study, we just focus on the origin-destination of each flight crossing the ECAC airspace. The specific network we will consider here refers to fligths occurring in day $1_{st}$ September 2017.

Applying the dismantling routines on this network, we find smaller differences among the different methods—$R_{ID}=0.1061$, $R_{IB}=0.0925, R_{CI2}=0.1093, R_{CI3}= 0.2783, R_{leiden}=0.01056 \pm 0.007$ (see Fig. 7. It is worth mentioning that when considering the Louvain and Infomap community detection methodologies we get average values $R_{louvain}=0.01060 \pm 0.005$ and $R_{infomap}=0.1057 \pm 0.0008$. The three different community detection algorithms give very close results, thus confirming the idea that the value of R seems to refer to a peculiar property of the network rather than of the way in which we partition it.

#### The email network

In what follows we replicate the dismantling analysis on a different dataset, usually used as a reference benchmark for community detection as it comes with ground truth community structure^36,37^.

The network has $N=1005$ nodes and $L=16687$ symmetric links distributed along 20 connected components whose largest size is 986. Nodes are researchers in a European institution and links are email sent between them. The given community structure reflect the partition in research departments. The dismantling analysis reveals a similar pattern to what already observed in the other datasets, with our community based approaching having a performance closer to IB than ID/CIs. Again, using the Louvain algorithm instead of Leiden gives similar results ($R_{leiden}=0.264\pm 0.002, R_{louvain}=0.264\pm 0.001$), while Infomap performs sensibly worse ($R_{infomap}=0.306 \pm 0.001$).

On this network we also considered a different partition in communities, namely the one given by the membership of the considered individuals (nodes) to one of the 42 departments. In this case we consider departments as communities and nodes are partitioned into 42 communities of sizes ranging from 2 to 109. When considering the Louvain methodology, the average largest size is $226 \pm 23$, the average smallest size is $56 \pm 7$ with an average number of communities which is about 8. When considering the Leiden methodology, the average largest size is $182 \pm 4$, the average smallest size is $32.7 \pm 0.5$ with an average number of communities which is again about 8. It is therefore clear that the partitioning in departments is much finer than those obtained with topology based community detection algorithms. However, the dismantling procedure based on the community structure given by the firm’s departments performs worse, with $R_{departments}=0.2822$. This might suggest that the information flow captured by the email exchange does not reflect the departmental structure of the firm. Rather, such flow is better captured by community detection algorithms based on the maximization of modularity.

#### The Facebook network

 We also investigated a network representing friendship connections on the Facebook social network^38^. The network has $N=4,039$ nodes and $L=88,234$ links, all belonging to a single giant connected component. On this network, we again find that IB provides the most efficient dismantling, followed by our approach. The approaches based on degree and CI all perform worse. Using Louvain instead of Leiden when retrieving the community structure we get $R_{louvain}=0.095 \pm 0.002$, again similar to $R_{leiden}=0.092 \pm 0.002$. Using Infomap the performance worsens, $R_{infomap}=0.116 \pm 0.001$.

#### The ca-CSphd collaboration network

 We also investigated a network representing scientific mentorship in the field of Computer Science^39^. Specifically, the links represent scientific ties between Ph.D. students and their advisors in theoretical computer science. The network has $N=1,882$ nodes and $L=1,740$ links distributed across 168 components. The largest connected component, on which we perform the dismantling has $N=1025$ nodes and $L=1043$ links. We find that this network is relatively easy to dismantle because of its relatively small density. Specifically, we find that IB performs the best, followed by our approach and by ID/CI based ones. When using our community based approach, Leiden and Louvain perform similarly ($R_{leiden}=0.0078 \pm 0.0002$,$R_{louvain}=0.0078 \pm 0.0002$), while Infomap communities are less effective ($R_{infomap}=0.0091 \pm 0.0001$).

## Discussion

Network dismantling has a twofold importance in the research stream on complex networks. On one side, it is related to the theoretical investigation of the resilience and the percolation properties of complex networks, and as such it helps in shredding light on the structural properties of several real world systems^1,2^. On the other, it holds an operational relevance in more applied contexts of investigation and anti-terrorism agencies^15^. In this paper, we have expanded on existing work^19^ to show the effectiveness of a dismantling procedure based on the community structure of a network. In fact, as communities represent distinct subsets of the nodes of a network which are characterized by higher inner connectivity, prioritizing the removal of nodes that bridges different communities is an effective way of rapidly dismantling the network. Indeed, we show how such a strategy proves its effectiveness in several different scenarios. Specifically, we investigated the performance of our method on a class of synthetic benchmarks generated with an implanted community structure. By tuning the inner density of the implanted communities we verified that the performance of our method is better when the community structure is more pronounced (i.e. inner density of communities is higher). We also tested the method on different real world networks and found that its performance are always comparable to the dismantling approach based on interactive betweenness computation and significantly better than the one based on degree and/or collective influence. Moreover, we show how the procedure that we designed has an unavoidable degree of stochasticity, as it is based on non-deterministic algorithm of community detection. With respect to this, we find that in spite of the differences in the outcomes of distinct realizations of the dismantling procedure, the effectiveness of each realization is strongly robust and does not vary significantly. Thus, our approach is able to identify several strategies that require the removal of distinct sets of nodes but all share a similar effectiveness. From an operational point of view, this result has strong implication and we believe is the most important contribution of this work: it allows to change the nodes that need to be removed to actually dismantle a network, allowing to find sets of nodes that minimize the removal costs without deteriorating the global effectiveness of the strategy. The robustness of the results also across different community detection algorithms suggest that the critical threshold that quantify the percentage of nodes that need to be removed to dismantle the network might be a network property, rather than being related to the specific dismantling path. However, we find that the dismantling tends to perform better when modularity based community detection are used. We believe this is due to the fact the definition of modularity fits well the scope of dismantling: if the community structure is designed to maximize the cohesiveness of internal connections, the bridge nodes we identify are more effective in splitting distinct parts of the network. We leave a more extensive exploration of this concept to future work.

**Figure 9:**
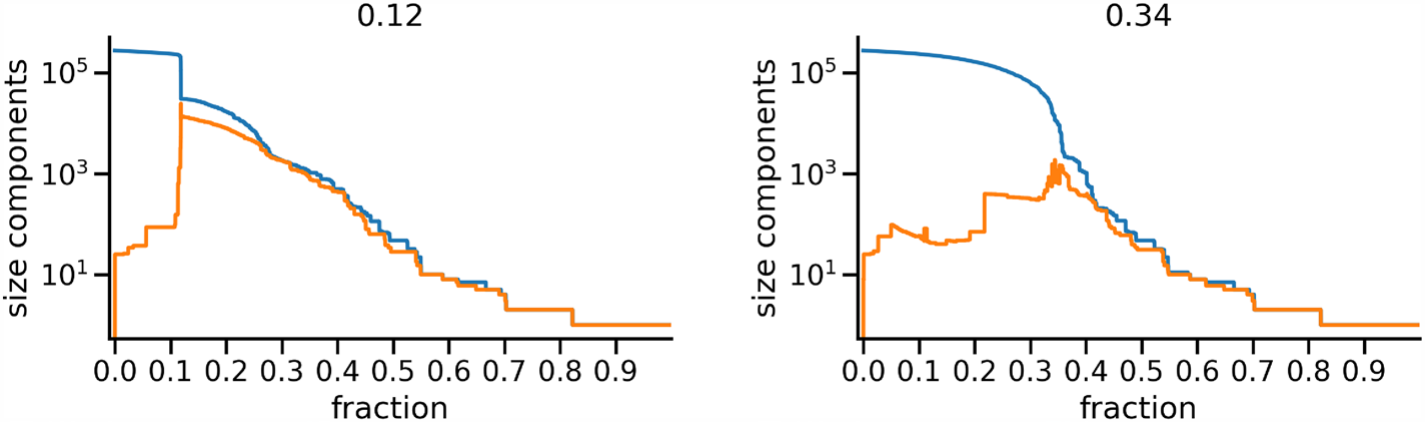
Sizes of the largest (LCC) and second largest (SLC) connected components as a function of the fraction of removed nodes for the complete IMDB network, using the community based strategy—Leiden algorithm (left panel) and the degree (right panel).

Our protocol always underperforms with respect to a node removal guided by the betweenness. This is true when considering different metrics such as the R metric as well as in terms of the percentage of nodes to be removed in order to have dismantling. This issue directly points to the question of what are the advantages of using our protocol. We try to answer by emphasizing three points:

1. When comparing with the betweenness, our protocol is certainly more performing in terms of computational times. This is especially true for large networks. In fact, let us consider again the network of male actors^40^ generated starting from data downloaded from the IMDB database. Without slicing such data according to the year of movie production, we get a large network made of 274,507 nodes and 6,408,592 links. In the left panel of Fig. 9 we show the size of the largest connected component (LCC) and second largest connected component (SLC) as a function of the fraction of nodes removed according to our protocol^15^. The critical threshold $q_c$ after which the networks gets dismantled is $q_c^{(comm)} \approx 0.12$. The right panel of the figure shows the same analysis for the case when the removal of nodes is done according to their degree. Although the behaviour is qualitatively the same, one can notice that the critical threshold after which the networks gets dismantled is $q_c^{(deg)} \approx 0.34$, which is significantly higher than $q_c^{(comm)}$. Our protocol needs 11 days on an Intel$\copyright$ based workstation Intel$\copyright$ Xeon$\copyright$ Gold 5118 CPU @ 2.30GHz - 2 processors - total 24 cores 512 Gb RAM. Similar results are obtained when using the R metric. We present here a percolation-based-like approach in order to emphasize that the result is not strictly linked to the use of R. The use of a removal protocol based on the iterative computation of the betweenness is not realistic. In this case the advantage of our methodology is clear.
2. When using protocols based on a removal of nodes guided by betweenness or degree, the ordered list of the nodes to be removed is fixed. In our protocol, when the community structure is extracted using stochastic algorithms, we can have different removal strategies all characterized by a similar efficiency. However, rather than being a flaw, we believe that this is a strength of our methodology. In fact, since we are interested in dismantling—and not in resilience—having different dismantling paths can be an advantage in all operational situations in which a specific set of nodes to be removed might be unreachable for several reasons, mainly related to their accessibility and/or the removal costs. However, when providing different strategies, we are interested in the stability of the percolation threshold at which dismantling occurs, which is related to the number of nodes that need to be removed from the network before considering it dismantled. In fact, we show that at least for the cases accounted for in this work, such threshold remains essentially the same on all possible strategies, notwithstanding the community detection algorithm. Moreover, we find that the effectiveness of our strategy depends on the significance of the community structure: in random networks, our strategy performs only slightly better than a degree based one.
3. In Ref.^15^ we showed how taking into account the modular structure of a network might be advantageous. Indeed, on lesson learnt by that case is that in a real world network advantages can not be merely measured in terms of computational efforts or percentages of nodes to be removed. In general, we want to back up the idea that the selection of the list of nodes to be removed might be guided by metadata that are related to its community structure. Therefore, simple strategies like the one proposed here may be effective in this respect. The method we propose here might also be further easily generalized by substituting the number (or percentage) of links with highest external degree with any other node attribute related to its community membership.

## Figures and Tables

**Figure 10:**
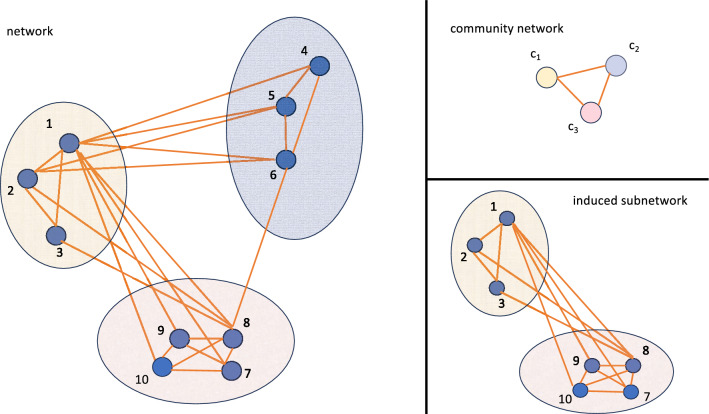
Worked example illustrating how Ref.^19^’s protocol might lead to different removal strategies with respect to ours. Explanations are given in the text of Appendix A.

## Data Availability

All the datasets used in our analysis are publicly available and properly referenced in the text, apart from the airport network that comes from a proprietary dataset that cannot be shared without authorization of the Eurocontrol authority. For any inquiry about the data please contact the authors at musci8@gmail.com

## Appendix A. Comparison with the methodology of Ref.^19^

Below we show a pedagogical example of how Ref.^19^’s protocol might lead to different removal strategies with respect to ours. Let us consider the configuration reproduced in the Fig. 10 below. When using our protocol, node 1 would be immediately selected and removed from the network together with its 9 links. When considering the protocol of Ref.^19^, first we have to consider the community network reproduced in the top-right side of the figure. In this network nodes $c_1$, $c_2$ and $c_3$ have degree 2, 1, 1, respectively. Following the example of Fig. 1 of Ref.^18^, we decide to consider the strategy by which we start attacking the community with largest size, i.e. community $c_3$. We have now to decide to adopt a strategy for ranking the links in the community network so that we can then decide how to select the links to remove from the community network. If we decide to follow strategy 1 of STEP 3 we need considering that $D_{i,j}=4$ for all the three links. If we decide to follow strategy 2 of STEP 3 we need considering that $S_{1,2}=9$, $S_{1,3}=12$, $S_{2,3}=12$. In both cases, we have degeneracies, i.e. there are equally ranked links. We therefore make the following choice: follow strategy 2 and randomly select what is the link to start with. Suppose that the random selection indicated that we have to start removing the link between community node $c_1$ and community node $c_3$. Let us now proceed to understand which nodes in the original network have to be removed (STEP 4). The receipt would require that we extract all inter-community links between community $c_1$ and community $c_3$ from the original network and then nodes in the induced subnetwork are then attacked by degree in decreasing order for simplicity and efficiency considerations. We therefore notice that both node 1 and node 8 have degree 6. We are therefore left with the doubt of removing node 1 or node 8.

We do not question on the fact that it is better to remove node 1 rather than node 8. We here simply want to emphasize that Ref.^19^ protocol might lead to different removal strategies with respect to ours. Moreover, let us consider a network equal to the previous one with the only presence of a further node 11 in community c3 that is only linked to both nodes 7, 8, 9 and node 10. In this case we should select node 8 for removal, without any ambiguity. However, such choice would have been guided by the presence of a node (exactly node 11) which has no role in bridging different communities. Again, we do not claim that our method is superior to the one of Ref.^19^: we are sure that any other scholar might detect flaws in our protocol as well. However, we think that the node selection for removal is more “transparent” in our protocol.

## Code availability

The code for replication of the results of the paper is available at https://github.com/musci8/DismantlingCommunity.

## References

[1] Cohen R, Erez K, Ben-Avraham D, Havlin S (2000). Resilience of the internet to random breakdowns. Phys. Rev. Lett..

[2] Albert R, Jeong H, Barabási A-L (2000). Error and attack tolerance of complex networks. Nature.

[3] Callaway DS, Newman ME, Strogatz SH, Watts DJ (2000). Network robustness and fragility: Percolation on random graphs. Phys. Rev. Lett..

[4] Albert R, Barabási A-L (2002). Statistical mechanics of complex networks. Rev. Mod. Phys..

[5] Kitsak M, Gallos LK, Havlin S, Liljeros F, Muchnik L, Stanley HE, Makse HA (2010). Identification of influential spreaders in complex networks. Nat. Phys..

[6] Morone F, Makse HA (2015). Influence maximization in complex networks through optimal percolation. Nature.

[7] Morone F, Min B, Bo L, Mari R, Makse HA (2016). Collective influence algorithm to find influencers via optimal percolation in massively large social media. Sci. Rep..

[8] Braunstein A, Dall’Asta L, Semerjian G, Zdeborová L (2016). Network dismantling. Proc. Natl. Acad. Sci..

[9] Gao J, Barzel B, Barabási A-L (2016). Universal resilience patterns in complex networks. Nature.

[10] Ren X, Gleinig N, Helbing D, Antulov-Fantulin N (2019). Generalized network dismantling. Proc. Natl. Acad. Sci..

[11] Grassia M, De Domenico M, Mangioni G (2021). Machine learning dismantling and early-warning signals of disintegration in complex systems. Nat. Commun..

[12] Zitnik M, Sosic R, Feldman MW, Leskovec J (2019). Evolution of resilience in protein interactomes across the tree of life. Proc. Natl. Acad. Sci..

[13] Solé RV, Rosas-Casals M, Corominas-Murtra B, Valverde S (2008). Robustness of the European power grids under intentional attack. Phys. Rev. E.

[14] Li D, Fu B, Wang Y, Lu G, Berezin Y, Stanley HE, Havlin S (2015). Percolation transition in dynamical traffic network with evolving critical bottlenecks. PNAS.

[15] Musciotto F, Miccichè S (2022). Effective strategies for targeted attacks to the network of Cosa Nostra affiliates. EPJDataScience.

[16] Ficara A, Curreri F, Fiumara G, De Meo P, Liotta A (2022). Covert network construction. Disrupt. Resilience Surv. Math..

[17] Fortunato S, Hric D (2016). Community detection in networks: A user guide. Phys. Rep..

[18] Requião da Cunha B, González-Avella JC, Goncalves S (2015). Fast fragmentation of networks using module-based attacks. PloS One.

[19] Wandelt S, Shi X, Sun X, Zanin M (2020). Community detection boosts network dismantling on real-world networks. IEEE Access.

[20] Morone F, Makse H (2015). Influence maximization in complex networks through optimal percolation. Nature.

[21] Karrer B, Newman MEJ (2021). Stochastic blockmodels and community structure in networks. Phys. Rev. E.

[22] Traag VA, Waltman L, van Eck NJ (2019). From Louvain to Leiden: Guaranteeing well-connected communities. Sci. Rep..

[23] Morone F, Min B, Bo L (2016). Collective influence algorithm to find influencers via optimal percolation in massively large social media. Sci. Rep..

[24] Blondel VD, Guillaume J-L, Lambiotte R, Lefebvre E (2008). Fast unfolding of communities in large networks. J. Stat. Mech. Theory Exp..

[25] Igraph betweenness: https://igraph.org/python/api/0.9.7/igraph._igraph.GraphBase.html#betweenness (2022).

[26] Leiden algorithm: https://github.com/vtraag/leidenalg (2022).

[27] Schneider CM, Moreira AA, Andrade JS, Havlin S, Herrmann HJ (2011). Mitigation of malicious attacks on networks. PNAS.

[28] Rosvall M, Bergstrom CT (2008). Maps of random walks on complex networks reveal community structure. Proc. Natl. Acad. Sci..

[29] Wandelt S, Sun X, Feng D (2018). A comparative analysis of approaches to network-dismantling. Sci. Rep..

[30] Tumminello M, Micciché S, Lillo F, Piilo J, Mantegna RN (2011). Statistically validated networks in bipartite complex systems. PLoS ONE.

[31] Gurtner G, Vitali S, Cipolla M, Lillo F, Mantegna RN, Miccichè S, Pozzi S (2014). Multi-scale analysis of the European airspace using network community detection. PLoS ONE.

[32] Bongiorno C, Gurtner G, Lillo F, Mantegna RN, Micciché S (2017). Statistical characterization of deviations from planned flight trajectories in air traffic management. J. Air Transport. Manag..

[33] Pappalardo, G., Mantegna, R. N. & Miccichè, S. ADAPT deliverable D5.1: Methodology for the empirical investigation of aircraft trajectories. https://adapt-h2020.eu/beta/wp-content/uploads/2019/09/783264-ADAPT-D5.1-Methodology-for-the-empirical-investigation-of-aircraft-trajectories-V01.01.00.pdf (2022).

[34] Milazzo M, Musciotto F, Micciché S, Mantegna RN (2022). Analysis of the structure and dynamics of European flight networks. Entropy.

[35] DDR2 Reference Manual For General Users 2.9.5. Eurocontrol, 2.9.5 edition (2018).

[36] Yin, H., Benson, A. R., Leskovec, J. & Gleich, D. F. Local higher-order graph clustering. In *Proceedings of the 23rd ACM SIGKDD International Conference on Knowledge Discovery and Data Mining,* 555–564 (2017). 10.1145/3097983.3098069 PMC5951164 29770258

[37] Email dataset: https://snap.stanford.edu/data/email-Eu-core.html (2023).

[38] McAuley, J. & Leskovec, J. *Learning to Discover Social Circles in Ego Networks* (NIPS, 2012).

[39] Johnson DS (1984). The genealogy of theoretical computer science. SIGACT News.

[40] Considering both the male and female part of the dataset was unfeasible for our computational tools.

